# The Coming-Out Process in Family, Social, and Religious Contexts Among Young, Middle, and Older Italian LGBQ+ Adults

**DOI:** 10.3389/fpsyg.2020.617217

**Published:** 2020-12-07

**Authors:** Fausta Rosati, Jessica Pistella, Maria Rosaria Nappa, Roberto Baiocco

**Affiliations:** ^1^Department of Developmental and Social Psychology, Faculty of Medicine and Psychology, Sapienza University of Rome, Rome, Italy; ^2^Department of Law, Economics and Human Sciences, Mediterranea University of Reggio Calabria, Reggio Calabria, Italy

**Keywords:** LGBQ+, generations, coming out, religion, Catholic, minority stress

## Abstract

The coming out (CO) process is fundamental for identity integration among LGBQ+ people, and its impact can vary greatly depending on personal and contextual factors. The historical, cultural, and social contexts in which LGBQ+ people develop their sexual identity can mediate the relationship between CO and health outcomes. The present study aimed at clarifying the CO process in three generations of Italian LGBQ+ people (young adults: aged 20–40 years; middle adults: aged 41–60 years; older adults: aged 61–80 years) by providing data on: (a) sexual orientation milestones, such as age of first awareness, age of first self-label, and age of first CO, as well as the rate of disclosure during different life stages; (b) the rate and average age of CO to significant others; and (c) CO within the religious context and its effect on participants’ minority stress experiences. A total of 266 Italian LGBQ+ people participated in the study, with ages ranging from 20 to 80 years (*M* = 41.15, *SD* = 16.13). Findings indicated that, on average, the older adult group became self-aware, self-labeled, and disclosed their sexual identity at a significantly older age than the other groups. Older adults were also more Catholic and had CO more frequently to their Catholic community, relative to young and middle adults. CO within the Catholic context was associated with distal and proximal minority stressors, such as discrimination, vigilance, and internalized sexual stigma. Catholic community reactions to participants’ CO were distinguished through thematic analysis in three main types: unconditional acceptance, invitation to change, and open rejection. The present research extended current knowledge on CO and minority stress experiences in different generations of LGBQ+ people. Several differences emerged between generational groups on sexual orientation milestones, highlighting the potential impact of historical and cultural contexts in determining sexual minorities’ experiences related to sexual identity. It is recommended that mental health professionals working with LGBQ+ clients implement targeted interventions based on their clients’ multiple salient aspects, including age and religious background. Clinicians should also be aware of the potentially detrimental effects of CO within an unsupportive context, rather than encouraging CO *tout court*.

## Introduction

Lesbian, gay, bisexual, queer, and other non-heterosexual (LGBQ+) people are consistently exposed to cis-heteronormative and cis-heterosexist pressures to fit heterosexual and binary gender roles. Cis-heteronormativity and cis-heterosexism are interrelated terms, the first referring to the assumption that all people are heterosexual and their gender identity matches with their birth-assigned sex (cis-gender), the latter indicating a shared beliefs system according to which heterosexual/cisgender people are considered more natural, real, and authentic than non-heterosexual/trans people (Serano, 2007). Consequently, trans/non-heterosexual people must come out to be recognized and become visible, while cisgender and heterosexual people do not have to define who they are because it is assumed that their identity and relationship experience is the norm. For these pressures and beliefs system, coming out (CO) – the act of disclosing one’s sexual orientation or gender identity to others – appears as one of the most stressful and pivotal experiences faced by LGBQ+ people (Cass, 1979). The visibility that results from the CO process may generate both benefits and costs, by protecting against or intensifying the effect of minority stressors. Although recent legal, cultural, and social changes in Western societies have generally improved the quality of sexual minorities’ identity development, only few studies have explored generational differences and similarities in the CO process (Jenkins Morales et al., 2014; Bishop et al., 2020). A relevant dimension needing further investigation in the literature is CO in the religious contexts, comparing the experiences of young, middle, and older LGBQ+ adults (Vaccaro, 2009). However, it is reasonable to assume that religiosity could affect the CO process in a positive, negative, or neutral way, depending on the individual’s age since older adults are generally more involved in religious activities compared to young adults (Chatters and Taylor, 1989). Age and religiosity may interact with the CO process at least at two main levels: (1) LGBQ+ older adults are more likely (currently and in the past) to be part of religious contexts and, therefore, they may feel a greater need to come out in such contexts than their younger counterparts; (2) the majority of LGBQ+ older adults started the CO process inside the religious contexts some years ago in a period in which Church and the whole society were more negative regarding sexual and gender minorities: In such hostile environments, LGBQ+ older were more likely to receive negative reactions to CO process than the new generations of LGBQ+ people (Dahl and Galliher, 2012).

Meyer (2003) conceptualized the sources of stress experienced by LGBQ+ people as *minority stressors*, as such stressors are linked to stigmatized social categories. The impact of minority stress can vary greatly, depending on other social categories that constitute identity, such as ethnicity, religion, gender, class, and age (Frost et al., 2019). Including both distal and proximal processes, minority stressors can be categorized into the following groups: (a) discrimination and/or harassment, experienced through external and objective events (Rollè et al., 2018); (b) vigilance, caused by an expectation of negative events; and (c) internalized sexual stigma (ISS), consisting of the internalization of negative attitudes and beliefs toward the self due to one’s LGBQ+ identity.

LGBQ+ aging people are generally considered as a particularly at-risk subgroup among the overall LGBQ+ population (Rosenfeld, 1999; Shankle et al., 2003; Dentato et al., 2014; Rosati et al., 2018, 2020). Their vulnerability can be explained considering the intersection of cis-heterosexism, the particularly hostile historical context in which they grew up, and ageism, that refers to the set of negative attitudes toward aging, including individual and institutional practices that perpetuate stereotypes, prejudice, and discriminatory practices toward older people (Butler, 1969). However, the few studies that have empirically compared different generations of LGBQ+ people have found higher levels of wellbeing and lower levels of minority stressors (e.g., harassment, rejection, and ISS) in LGBQ+ older adults, compared to younger adults (Cortes et al., 2019; Wickham et al., 2019). Vaccaro (2009) found more similarities than differences when comparing three generations of sexual minorities regarding the CO process, family reactions to CO, activism, and discrimination. In a study interested in examining parental responses to CO in three cohorts of LGBQ+ people, emerged that the youngest cohort was more likely to experience validating responses, however, invalidating responses were frequent across all cohorts without differences (van Bergen et al., 2020). Furthermore, in comparing two generations of LGBQ+ adults, Jenkins Morales et al. (2014) found that the younger group presented a worse perception of legal and healthcare access, less community involvement, and higher rates of verbal harassment compared to the older group. The authors explained these findings as the consequence of younger adults’ higher disclosure of sexual identity–a sign of identity affirmation, but also a factor known to increase the risk of stigma and victimization.

Indeed, although the CO process is fundamental for LGBQ+ people’s identity integration (Cass, 1979; LaSala, 2000), the act of disclosing one’s sexual identity to others can be an important source of stress. Postmodern and feminist theories have questioned the essentialist concept of CO as a linear path involving universal or prescribed stages and described sexual identity development as shaped by historical era and social context (Rust, 1993; Broido, 2000). From this perspective, CO is not conceptualized as a single event, but rather as a non-linear path involving different relationships and contexts which strongly influence the quality of the experience (Gusmano, 2008). Perhaps for this reason, the findings of studies investigating the effect of CO on wellbeing have been quite controversial. On the one hand, CO has been recognized as fundamental for improving self-esteem, life satisfaction, and the quality of relationships (Savin-Williams, 1989; Monroe, 2001; Rosario et al., 2001; Heatherington and Lavner, 2008); on the other hand, greater visibility following disclosure has been found to be associated with higher victimization (D’Augelli and Grossman, 2001). Similarly, while some studies have identified the concealment of one’s sexual identity as a dysfunctional coping strategy for both physical (Cole et al., 1996) and mental health outcomes (Morris et al., 2001), others have found a lack of influence (Fredriksen-Goldsen et al., 2013)—or even a protective role (Cole, 2006)—of concealment on health indicators.

Several studies have revealed the importance of social and contextual variables in determining the relationship between CO and wellbeing, highlighting the harmful impact of the negative reactions of significant persons, such as parents (D’Augelli et al., 1998; Willoughby et al., 2006; Baiocco et al., 2015; Baiocco and Pistella, 2019), siblings (Pistella et al., 2020a), and close friends (Ryan et al., 2015). Legate et al. (2012) showed that CO was associated with more positive wellbeing when it occurred in supportive contexts, whereas this association was not present in controlling contexts–such as religious ones. Although religion is generally associated with positive psychosocial outcomes (Cotton et al., 2006), sexual minority people may feel (or be) rejected by their religious community, or stop practicing a religion altogether, due to a perceived conflict with their sexual minority status. Through a qualitative investigation, Dahl and Galliher (2012) found that CO in religious environments could lead to both positive and negative outcomes, with the latter including feelings of inadequacy, religious-related guilt, depressive symptoms, and social strain. Additionally, high family religiosity has been found to be strongly associated with parents’ rejection of LGBQ+ children (Baiocco et al., 2015; Snapp et al., 2015; Heiden-Rootes et al., 2019, 2020).

The role of religion in sexual minorities’ wellbeing is still not clear, with studies reporting it as a positive resource in the lives of many LGBQ+ individuals (Rosenkrantz et al., 2016), while others indicating it as a risk factor for experiencing ISS (Lingiardi et al., 2012; Severson et al., 2014; Sowe et al., 2014; Nardelli et al., 2020). This lack of coherence in literature may depend on the fact that not all religious contexts are stigmatizing (Coley, 2017), and that some LGBQ+ people succeeded in reconciling their faith with their sexual identity (Beagan and Hattie, 2015). In Italy, where the present research was conducted, the most practiced religion is Catholicism, and there is a lack of openly inclusive LGBQ+ contexts. This could be due to the fact that the Italian Catholic Church – as Italian culture in general – is strongly based on traditional values (e.g., clear division of gender roles) and conservative religious beliefs, thus representing a potentially dangerous environment for Italian sexual minorities. In fact, LGBQ+ people who belong to non-affirming religious communities (Barnes and Meyer, 2012) or who use negative religious coping (Brewster et al., 2016) are highly at risk of experiencing ISS. With regard to the other minority stressors, to our knowledge, no prior study has investigated the expectation of negative events (i.e., vigilance) among LGBQ+ persons who belong to a religious community, and only a few studies have considered experiences of discrimination from one’s religious community; these studies have found such discrimination to relate to higher ISS and greater religious struggle, which, in turn, were associated with poorer wellbeing (Szymanski and Carretta, 2020).

### Present Study

In Italy, cis-heterosexism and cis-heteronormativity is pervasive at an institutional level, and LGBQ+ people face stigma and prejudice in several contexts (Baiocco and Pistella, 2019; Rollè et al., 2020). Previous research has identified the family, school, and healthcare arenas as potentially negative environments for Italian sexual minorities (Baiocco et al., 2015, 2020; Rosati et al., 2020), and other research has highlighted the relationship between ISS and CO (Baiocco et al., 2016; Pistella et al., 2020b). As mentioned above, another environment potentially causing minority stress for Italian sexual minorities is represented by the Catholic Church: a recent study (Garelli, 2013) estimated that approximately 80% of Italian citizens identify as Catholic, thus for sure including also a share of the Italian LGBQ+ population. For instance, some Italian LGBQ+ people are members of LGBQ+ Catholic associations, whose aim is precisely to tackle stigma against sexual minorities in a Catholic environment and to support the reconciliation of faith and sexual identity.

In order to gain insight into the CO experiences of three generations of Italian LGBQ+ people (young adults: aged 20–40 years; middle adults: aged 41–60 years; older adults: aged 61–80 years), the present study aimed at: (a) providing descriptive data on sexual orientation milestones, such as the age of first awareness, self-labeling, and CO, and the rate of disclosure at different life stages; (b) providing descriptive data on meaningful features of CO, such as the rate and average age of first disclosure to family members, friends, coworkers, neighbors, and family doctors; and (c) examining the CO process within the religious context and its effect on participants’ experiences of minority stress. A further aim of the study was to explore the quality of the reactions that LGBQ+ people received from their religious community in response to their CO.

## Materials and Methods

### Procedures and Participants

Recruitment occurred through purposive and snowball sampling, beginning with the first author’s personal contacts. Flyers were also posted on social media and within LGBTQ+ centers/meeting places. Inclusion criteria were: (a) having lived in Italy for at least 20 years; (b) self-identified as LGBQ+; and (c) aged 20–80 years. Before data collection began, the research protocol was approved by the Ethics Commission of the Department of Developmental and Social Psychology, Sapienza University of Rome. The survey was then uploaded online. Participants first gave their consent to the research before accessing the rest of the questionnaire, which took them, on average, 30 min to complete. From the original sample (*n* = 291), 11 participants were excluded because they self-identified as heterosexual. Again, for the purpose of this study, we did not consider 14 participants whose stated religion was non-Catholic (3% Buddhist, 6% Rastafarian, 3% Pagan, 1% Jewish, and 2% Waldensian), due to the small number of participants per religion and the religions’ differing conceptions of LGBQ+ issues.

The final sample was comprised of 266 Italian LGBQ+ people, aged 20–80 years (*M* = 41.15, *SD* = 16.13). In accordance with previous research (Howe and Strauss, 1992; Vaccaro, 2009), participants were divided into generational groups, as defined by certain historical and cultural events (e.g., the post-war period, civil rights movements, the technological revolution). Ultimately, we considered three generations of LGBQ+ people: young adults (aged 20–40 years; *n* = 145), middle adults (aged 41–60 years; *n* = 61), and older adults (aged 61–80 years; *n* = 61), which, respectively, corresponded to *millennials* (born after 1981), *Generation Xers* (born between about 1960 and 1980), and *baby boomers* (born between about 1940–1960) (Howe and Strauss, 1992; Vaccaro, 2009). Most participants (94%) self-identified as cisgender men (*n* = 123; 46%) and women (*n* = 126; 47%), while 6% (*n* = 17) self-identified as transgender/non-binary/genderqueer.

Concerning sexual orientation, 77% of the women identified as lesbian, 20% as bisexual, and 3% as queer, pansexual, or fluid; among the men, 97% self-identified as gay and 3% as bisexual; most transgender/non-binary participants self-identified as queer, pansexual, or fluid (86%), with the remaining 14% as bisexual. Table 1 presents data on the sexual orientation of all participants and the different age groups. Regarding ethnicity, most participants (95%) were White, 3% were Hispanic, and 2% were Asian. More than half of the participants (72%) reported an average socio-economic status, whereas 17% reported a low status and 11% reported a high status. Educational level varied from high school diploma (39%) to bachelor’s or higher degree (61%).

**TABLE 1:** Rates of sexual orientation labels in young, middle, and older LGBQ+ adults.

	Young (*n* = 145) *n* (%)	Middle (*n* = 61) *n* (%)	Older (*n* = 60) *n* (%)	Total (*n* = 266) *n* (%)
Lesbian	54 (40.7%)	27 (44.3%)	22 (56.7%)	103 (38.7%)
Gay	59 (37.2%)	25 (41.0%)	34 (36.7%)	118 (44.4%)
Bisexual	18 (12.4%)	4 (6.6%)	3 (5.0%)	25 (9.4%)
Queer/Pansexual/Fluid	14 (9.7%)	5 (8.2%)	1 (1.7%)	20 (7.5%)

### Measures

#### Sociodemographic Variables

The survey included several sociodemographic questions to obtain information on participants’ age, gender identity, sexual orientation, ethnicity, socio-economic status, and education level. Participants indicated their sexual orientation from one of six options: heterosexual, mainly heterosexual, bisexual, mainly gay/lesbian, gay/lesbian, and other (with the request to specify).

#### Sexual Orientation Milestones

To obtain information on participants’ experiences related to the development of their sexual orientation, we used several items from D’Augelli and Grossman (2001). Specifically, participants were asked at what age: (a) they became aware that they were attracted to people of the same gender (i.e., age of first awareness); (b) they started using a “label” to describe their sexual orientation (i.e., age of first self-label); and (c) they first told someone about their sexual orientation (i.e., age of first CO). Moreover, experiences of CO to specific figures (i.e., mothers, fathers, siblings, children, nephews, grandparents, best friends, employers, co-workers, neighbors, family doctor) were also investigated. Participants were asked to specify whether they had CO to each of these figures and, if so, to specify the age of disclosure; they were also asked to indicate if they had not yet CO to each figure or if the situation was not applicable (e.g., if the respondent did not have children). Finally, we investigated the percentage of figures who knew about the participant’s sexual orientation during the participant’s adolescence (13–18 years old), emerging adulthood (19–30 years old), and adulthood (31–59 years old), and at the present time (i.e., the time of study). Obviously, for the young adult and middle adult groups, we did not consider answers referring to an age of CO that was not applicable (e.g., for a young adult aged 29 years we considered only the percentage of figures who knew about the participant’s sexual orientation during the participant’s adolescence and emerging adulthood, and at the present time).

#### Religious Variables

Religiosity was evaluated using both quantitative and qualitative procedures. Participants were asked to indicate whether they followed a religion (0 = yes; 1 = no), as well as to specify which religion they followed (through an open-ended question). Additionally, religious participants were asked: “Have you ever talked about your sexual orientation with priests or nuns or other members of your religious community?” (0 = yes; 1 = no; 2 = I do not belong to a religious community). Participants who answered affirmatively were then asked to describe the reaction of their religious community to their CO, through an open-ended question: “What were their reactions and how did you feel about that?”

#### Minority Stressors

Three measures were used to investigate the sources of minority stressors–both distal and proximal–identified as relevant to the LGBQ+ community (Meyer, 2003): (a) experiences of discrimination/harassment, (b) vigilance, and (c) ISS. Two subscales of six items each were taken from the Daily Heterosexist Experiences Questionnaire (DHEQ; Balsam et al., 2013) to assess experiences of discrimination and vigilance. Further to this, participants were asked the following question: “How much has this problem distressed or bothered you during your life?” They registered their response to this on a six-point Likert scale ranging from 0 (*did not happen/not applicable to me*) to 5 (*it happened, and it bothered me extremely*).

The DHEQ Discrimination/Harassment subscale was used to assess participants’ experiences of external, objective stressful events (distal minority stressors). Example items were: “Being called names, such as fag or dyke” and “People laughing at you or making jokes at your expense because you are LGBQ+.” Cronbach’s α for the Discrimination/Harassment subscale score was 0.80. The DHEQ Vigilance subscale was used to measure expectations of homo-lesbo-biphobia attitudes, which often lead LGBQ+ people to conceal their sexual identity (distal-proximal minority stressor). Example items were: “Watching what you say and do around heterosexual people” and “Hiding your relationship from other people.” Cronbach’s α for the Vigilance subscale score was 0.87.

The Measure of ISS for Lesbians and Gay Men–Short Version (MISS-LG; Lingiardi et al., 2012) was used to measure the internalization of homo-lesbo-biphobia, which manifests as negative attitudes held by LGBQ+ people toward non-heterosexual sexual orientation and, accordingly, toward themselves (proximal stressor). Example items were: “When I have sex with someone of the same gender, I feel awkward” and “I do not believe in love between LGBQ+ people.” Participants were asked to express their agreement with each item on a five-point Likert scale ranging from 1 (*strongly disagree*) to 5 (*strongly agree*), with higher scores indicating higher levels of ISS. Cronbach’s α for the total score was 0.82.

### Data Analysis

The Statistical Package for the Social Sciences (SPSS 25.0) was used to conduct the quantitative analysis. The chi-square test was used to investigate differences between groups in terms of the rate of CO to relevant figures and religiosity. Univariate analyses of variance (ANOVAs) were used to analyze group differences regarding sexual orientation milestones and the average age of CO to significant figures. A multiple analysis of variance (MANOVA) was used to assess the effect of generations and CO in a religious context on minority stress indicators.

Finally, thematic analysis (Braun and Clarke, 2006) was used to identify the main categories within the qualitative data. Specifically, a theoretical approach driven by the research area of interest was adopted at a semantic level, when interpreting participants’ answers. This analysis consisted in several steps, involving the familiarization of all authors with the data, followed by a discussion of the first emerging contents. Once the main thematic categories were identified, the first author re-coded, where necessary, all transcripts to align them with the correspondent theme.

## Results

### Sexual Orientation Milestones

**FIGURE 1:**
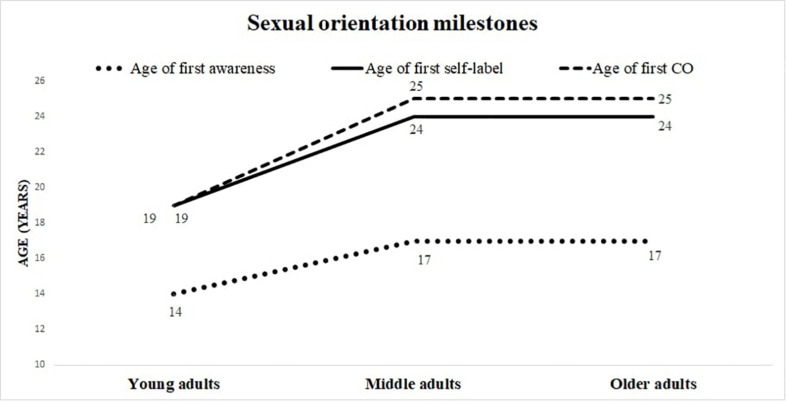
Sexual orientation milestones.

Participants reported becoming aware of their sexual orientation around the age of 15 years, and first self-labeling and disclosing their sexual identity around the age of 21 years (Table 2). Young adults became aware of, self-labeled, and disclosed their sexual identity at a significantly younger age than did middle and older adults (Figure 1). The percentage of people who knew about participants’ sexual orientation during different life stages was approximately 17% during adolescence, 54% during emerging adulthood, 69% during adulthood and 76% at the present time. The three groups significantly differed in their rates of disclosure during adolescence and emerging adulthood, with young adults demonstrating greater disclosure than the other two groups, and middle adults presenting significantly greater discloser than the older adults. No significant differences were found between groups in the rate of disclosure during adulthood and at the present time. Specifically, young adults were known to be LGBQ+ by 24, 68, 73, and 78% of others, during middle adolescence, emerging adulthood, adulthood, and the present time, respectively; comparable others for middle adults were 10, 43, 72, and 74%; and for older adults, 7, 34, 62, and 76%.

**TABLE 2:** Sexual orientation milestones in young, middle, and older LGBQ+ adults.

	Total Sample (*n* = 266) *M* (SD)	Young adults (*n* = 145) *M* (SD)	Middle adults (*n* = 61) *M* (SD)	Older adults (*n* = 60) *M* (SD)	*F*	*p*
Age of first awareness	15.17 (7.53)	13.99 (5.60)	16.61 (8.53)	16.63 (9.95)	4.06	<0.05
Age of first self-label	21.39 (7.29)	19.13 (4.56)	24.43 (8.62)	24.08 (9.39)	17.90	<0.001
Age of first CO	21.83 (6.97)	19.26 (4.48)	24.95 (8.02)	25.24 (8.31)	26.95	<0.001
% of CO in adolescence	17.26 (26.94)	24.31 (29.45)	10.25 (25.16)	7.2 (14.72)	12.08	<0.001
% of CO in emerging adulthood	54.29 (31.15)	67.61 (24.51)	43.03 (31.67)	33.9 (29.64)	37.98	<0.001
% of CO in adulthood	69.22 (29.01)	73.48 (27.34)	71.72 (27.19)	61.86 (31.61)	2.89	0.06
% of current CO	76.79 (21.96)	78.45 (19.23)	73.77 (23.90)	75.85 (25.83)	1.04	0.35

*The *F* refers to the difference between young, middle, and older adults. Adolescence, 13–18 years; Emerging adulthood, 19–30 years; Adulthood, 31–59 years. Totals vary in variable “% of CO in adulthood”, because only participants having at least 31 years were considered.*

### Disclosure of Sexual Orientation to Significant Others

Table 3 reports participants’ rates and average ages of disclosure to relevant figures, as well as the χ^2^ and *p-*values for each variable. Most participants reported having disclosed their sexual orientation to their mother (76%), father (59%), and siblings (84%). Young adults were significantly more disclosed to both their mother (85%) and their father (71%) than were older adults (CO to mother: 47%; CO to father: 18%), whereas no significant difference was observed between young adults (88%), middle adults (93%), and older adults (75%) in the rate of CO to siblings. Among the participants with children, more than one-fourth (35%) had CO to them. Only one participant in the young adult group with children had not CO to them, whereas 44% of the middle adults and 50% of the older adults had disclosed their sexual identity to their children. No chi-square test was run to compare the three generations, due to the small number of participants per cell-group (<5). Almost half (44%) of the participants had CO to their nephews, with no significant difference found between the young adults (35%), middle adults (53%), and older adults (44%). Only 19% of the total sample had CO to their grandparents (21% young adults; 18% middle adults; 12% older adults). A chi-square test was not applicable.

**TABLE 3:** Differences between young, middle, and older LGBQ+ adults in rate and average age of coming out (CO) with significant others.

	Total Sample (*n* = 266)	Young adults (*n* = 145)	Middle adults (*n* = 61)	Older adults (*n* = 60)		
				
	*n* (%) Age (*M*, SD)	*n* (%) Age (*M*, SD)	*n* (%) Age (*M*, SD)	*n* (%) Age (*M*, SD)	χ*^2^ F*	*p(*χ*^2^) p(F)*
Coming out to mother	191 (76.1%) 24.76 (9.12)	121 (84.6%) 21.83 (6.6)	47 (79.7%) 27.87 (8.01)	23 (46.9%) 33.83 (13.89)	29.01 25.61	<0.001 <0.001
Coming out to father	138 (58.7%) 23.71 (6.89)	99 (71.2%) 22.06 (5.89)	31 (60.8%) 27.87 (7.4)	8 (17.8%) 28 (8.42)	40.17 11.61	<0.001 <0.001
Coming out to siblings	183 (83.6%) 26.09 (10.2)	101 (87.8%) 21.41 (4.76)	43 (82.7%) 28.63 (8.34)	39 (75%) 35.41 (14.37)	4.32 40.58	0.11 <0.001
Coming out to children	11 (37.9%) 46.30 (10.54)	0 (0%) /	4 (44.4%) 43.33 (11.37)	7 (50%) 47.57 (10.83)	/ 0.31	/ 0.59
Coming out to nephews	45 (43.7%) 35.74 (12.38)	13 (35.1%) 25.85 (10.06)	16 (53.3%) 38.29 (6.28)	16 (44.4%) 41.56 (13.67)	2.24 8.42	0.33 <0.01
Coming out to grandparents	28 (18.8%) 24.79 (5.81)	19 (21.3%) 23.11 (4.63)	5 (17.9%) 30.8 (7.98)	4 (12.5%) 25.25 (3.4)	/ 4.36	/ <0.05
Coming out to best friend	250 (97.7%) 22.59 (8.18)	141 (98.6%) 19.38 (4.29)	60 (100%) 25.38 (8)	49 (92.5%) 28.39 (11.86)	8.26 33.59	<0.05 <0.001
Coming out to employer	114 (58.2%) 30.76 (9.14)	65 (62.5%) 26.31 (4.97)	31 (60.8%) 34 (7.93)	18 (43.9%) 42.56 (11.41)	4.37 38.63	0.11 <0.001
Coming out to colleagues	195 (83.7%) 29.05 (8.47)	106 (85.5%) 24.86 (4.41)	46 (82.1%) 31.96 (6.68)	43 (81.1%) 36.63 (11.3)	0.64 47.8	0.72 <0.001
Coming out to neighbors	57 (28.6%) 31.77 (11.04)	25 (22.7%) 25.48 (5.08)	21 (39.6%) 32.95 (9.61)	11 (30.6%) 45 (12.79)	5.07 18.63	0.08 <0.001
Coming out to family doctor	93 (39.7%) 30.41 (11.82)	43 (33.1%) 23.44 (4.98)	22 (40.7%) 31.14 (8.67)	28 (56%) 40.93 (13.79)	7.95 29.88	<0.05 <0.001

*The χ^2^ refers to the difference between young, middle, and older adults. The frequencies refer to the answer “He/she is aware of my sexual orientation” to the questions. The χ^2^ is not applicable (NA) for those variables with <20% of cells with expected frequencies <5. Totals for each variable vary because of missing data. The “/” refers either to a missing value due to the lack of participants; or to the impossibility to apply the χ^2^ variable.*

CO to a best friend emerged as the highest rate of disclosure, involving 98% of participants. Middle adults (100%) had CO to their best friend significantly more frequently than older adults (92%), whereas young adults (97%) fell between these two groups. Just over half of the participants (58%) had disclosed their sexual orientation to their employer, with no significant difference between generations (62% young adults; 61% middle adults; 44% older adults). A high percentage of participants (84%) had disclosed their sexual orientation to coworkers, with no differences between young adults (85%), middle adults (82%), and older adults (81%). CO to neighbors involved 29% of total sample (23% young adults; 40% middle adults; 31% older adults), with no significant difference between groups, χ^2^(1) = 5.074, *p* = 0.08.

Finally, 40% of participants had disclosed their sexual orientation to their family doctor, with significant differences between generations: more older adults (56%) had disclosed to their family doctor relative to young adults (33%). Regarding the average age of CO to different figures (Table 3), the best friend emerged as the first person to whom most participants disclosed their sexual orientation, at around 19 years of age for young and middle adults. Older adults, in contrast, CO first to grandparents at the age of 25 years, and then to a father and best friend at the age of 28.

A series of ANOVAs revealed significant differences between generations in the average age of CO, with young adults CO to significant others earlier than middle and older adults. Specifically, young adults CO to their mother, siblings, best friend, employer, coworkers, neighbors, and family doctor at an earlier age than middle adults; likewise, middle adults CO to these figures at an earlier age than older adults. Additionally, younger adults first disclosed their sexual orientation to their father and nephews at an earlier age than middle and older adults, who did not significantly differ in the average age of CO to these figures. Middle adults CO to grandparents at an older age (approximately 31 years) than young adults (23 years) and older adults (25 years). CO to children occurred at approximately 46 years of age, with no significant differences between middle and older adults.

### Rates of Catholicism and CO in the Religious Context

In our sample, 28% of participants self-identified as Catholic, whereas 72% self-identified as atheist. Older adults (42%) were significantly more religious than young adults (20%) (Table 4). More than half (67%) of the Catholic participants had CO to their religious community, with the older adults more disclosed (84%) than the middle adults (71%) and young adults (48%). Of those who had CO to their religious community, 60% were not accepted; older adults were the most rejected (71%), followed by young adults (57%), and middle adults (48%). As explained below, non-acceptance from the Catholic community could manifest as either an invitation to change or open rejection.

**TABLE 4:** Religious variables in young, middle, and older LGBQ+ adults.

	Total Sample (*n* = 266) *n* (%)	Young (*n* = 145) *n* (%)	Middle (*n* = 61) *n* (%)	Older (*n* = 60) *n* (%)	χ*^2^*	*p*
Catholic	75 (28.2%)	29 (20.0%)	21 (34.4%)	25 (41.7%)	11.36	<0.01
CO to Catholic community	50 (66.7%)	14 (48.3%)	15 (71.4%)	21 (84.0%)	8.01	0.01
Non-acceptance	30 (60.0%)	8 (57.1%)	7 (46.7%)	15 (71.4%)	2.30	0.32

*The χ^2^ refers to the difference between young, middle, and older adults. The frequencies, respectively, refer to the answers to the following questions: “Do you follow a religion?” (Q1); “Have you ever talked about your sexual orientation with priests or nuns or other member of your religious community?” (Q2); “If Yes, what were their reactions?” (Q3). The sample size varies in each variable, depending on answers to previous questions: only participants who answered “Yes” to Q1 and define their religion as Catholic (*n* = 75) were considered for Q2; only participants who answered “Yes” to Q2 (*n* = 50) were considered for Q3.*

To explore the characteristics of the Catholic community’s reactions to participants’ CO, we analyzed the content of participants’ descriptions, as well as their stated feelings in response to these reactions [i.e., in response to the open-ended question: “What were their reactions (priests, nuns, or other members of your religious community) and how did you feel about that?”]. Through the theoretical thematic analysis (Braun and Clarke, 2006), we identified three main types of reactions, ranging from acceptance to rejection (Table 5).

**TABLE 5:** Catholic community reactions to CO: Thematic categories and representative quotations (*n* = 50).

	Community behaviors	Associated feelings
Unconditional acceptance (*n* = 20: 6 young adults, 8 middle adults, 6 older adults)	*During a confession, Don Luigi told me: “What right do I have to judge you? The church should practice acceptance and in church you must feel welcome”* (64-year-old gay man)	*I felt wonderfully well*
	*In the Catholic Church I received complete acceptance by both the parish priest and the bishop of the diocese. They allowed me to be my partner’s godmother of confirmation* (33-year-old lesbian woman)	*I was very serene, it seemed to me a very normal thing*
Invitation to change (*n* = 12: 4 young adults, 5 middle adults, 3 older adults)	*Someone expressed understanding for the person but condemnation of the practice* (44-year-old gay man)	*I felt welcomed while being invited to change*
	*I came out during confessions or personal spiritual directions. Hardly anyone was scandalized, except for one priest who advised me to have sexual relations with men even outside of marriage* (42-year-old lesbian woman)	*On some occasions I felt protected, while on others I felt left out*
Open rejection (*n* = 18: 4 young adults, 2 middle adults, 12 older adults)	*They told me it was wrong, that it would be just a moment and that I had to pray to become normal* (39-year-old lesbian woman)	*Depressed, guilty, and humiliated as a person. I felt ashamed for who I was and tried to change my sexual orientation*
	*Someone proposed and/or subjected me to exorcisms or prayers. Someone told me that my attraction to women was due to the fact that I didn’t want to leave control of my life to a man and that this had to be changed* (31-year-old bisexual woman)	*Guilty, wrong, inadequate*
	*They told me I shouldn’t have sex, as if the issue was related to sex and not love* (79-year-old lesbian woman)	*I felt betrayed by my own religion. As if I no longer had reference points*

*Transcriptions of some answers to the following open-ended questions: (a) “Have you ever talked about your sexual orientation with priests or nuns or other members of your religious community? What were their reactions?” and “How did you feel about that?”*

*Unconditional acceptance* represented an extremely positive reaction to CO, mainly consisting of the interpretation and application of Catholic values such as love and a sense of welcoming. Participants who experienced unconditional acceptance were permitted to attend and/or actively participate in religious rituals, and experienced feelings of wellbeing, serenity, and psychological integrity.

*Invitation to change* can be considered as a neutral or ambiguous reaction, which can be well represented by the Catholic expression “hating the sin but not the sinner” (Valera and Taylor, 2011). When it comes to sexual orientation, separating the practice (loving and having sex with someone of the same gender) from the person is not possible and the person concerned risks to be trapped in an unclear position. Moreover, some problematic aspects underlie the apparent acceptance characterizing this reaction, thus increasing ambiguity, such as the encouragement of having sex with opposite-gender partners that some participants reported having received from their priests.

Finally, *open rejection* is the negative extreme of the Catholic community’s range of potential reactions to CO, characterized by an overt opposition to non-heterosexual identity and abusive prescriptions, often resulting in the application of “reparative therapeutic” techniques such as sexual abstinence, prayers, and exorcisms. The effects of these practices on participants’ emotional and mental condition include guilt, inadequacy, mistrust, humiliation, and depression.

### Impact of CO to the Catholic Community on Minority Stressors

Table 6 reports differences in levels of discrimination, vigilance, and ISS between participants who: (a) had not CO in a religious context due to the absence of a religious community (atheists), (b) had not CO to their religious community (concealed), and (c) had CO to their religious community (disclosed). To test the effect of CO in the religious context, we performed a MANOVA using age as a covariate. Compared to atheist participants, those who had disclosed their sexual orientation to the Catholic community showed higher levels of discrimination, vigilance, and ISS, whereas participants who had concealed their sexual orientation from the Catholic community had scores falling between those of these two groups. Age had a significant effect on discrimination and vigilance: the older the participant, the more likely they were to be subjected to more stressors; however, no association was found between ISS and age. No differences were found in the levels of minority stress by using generation as an independent variable.

**TABLE 6:** Means and standard deviations for discrimination, vigilance, and internalized sexual stigma (ISS) by CO to Catholic community.

	Discrimination			Vigilance			Internalized sexual stigma		
			
	*M* (SD)	*F*	*p*	*M* (SD)	*F*	*p*	*M* (SD)	*F*	*p*
**CO to Catholic Community**									
Disclosed	1.43 (1.40)	3.87	<0.05	1.9 (1.75)	4.64	<0.01	2.02 (1.67)	8.45	<0.001
Concealed	1.09 (0.97)			1.43 (0.75)			1.77 (0.63)		
Atheist	0.95 (1.01)			1.29 (1.15)			1.54 (0.63)		

*Significant effect of age (used as a covariate) emerged for discrimination and vigilance, but not for ISS.*

## Discussion

The present study aimed at expanding the empirical literature on the CO process, as experienced by different generations of LGBQ+ people, as well as verifying the effect of CO in understudied contexts, such as the religious context. Considering certain historical and cultural events as fundamental in shaping generational divides, we categorized participants into groups of young, middle, and older adults, corresponding to millennials, Generation Xers, and baby boomers, respectively (Howe and Strauss, 1992; Vaccaro, 2009).

Significant differences were found between generations on all sexual orientation milestones and almost all CO variables (Dunlap, 2016; Bishop et al., 2020). On average, young adults experienced self-awareness, self-labeling, and disclosure of their sexual identity 3–5 years earlier than middle and older adults (Figure 1). Considering the total sample, approximately 6 years elapsed between participants’ average age of first awareness, and the first time they self-label as LGBQ+ and CO, with young adults demonstrating a shorter period (approximately 5 years), and middle and older adults demonstrating a longer period (approximately 8 years). Additionally, young and middle adults were more likely than older adults to not recognize themselves in the provided sexual orientation categories (i.e., heterosexual, gay/lesbian, bisexual), and to instead use the open-ended answer option to identify themselves as queer, pansexual, fluid, etc. This is consistent with previous research reporting millennials’ tendency to reject traditional and normative labels of gender and sexual orientation (Russell and Bohan, 2005; Vaccaro, 2009).

As found in previous studies, the rate and average age of disclosure decreased as participant age increased (Jenkins Morales et al., 2014; Dunlap, 2016; Bishop et al., 2020; Pistella et al., 2020a). The earlier age at which young adults first became aware of and disclosed their sexual identity likely relates to the increased cultural references to and social acceptance of sexual minorities in Western countries, including Italy (Russell and Fish, 2016). Considering the total sample, participants’ average age of first CO was approximately 22 years, which is considerably later than that recorded in other countries (Dunlap, 2016; Russell and Fish, 2016). However, the average age of CO among young adults was 19 years, in line with prior national studies investigating participants in a similar age range (Barbagli and Colombo, 2007; Baiocco et al., 2015; Pistella et al., 2020a).

Accordingly, young adults were significantly more disclosed than older groups during adolescence (aged 13–18 years) and emerging adulthood (aged 19–30 years), whereas no meaningful differences emerged between groups during adulthood (aged 31–59 years) and the present time, suggesting that generations did not differ in their choice of CO (i.e., whether to CO), but only in their process of CO (i.e., when to CO) (Dunlap, 2014). At the present time, participants’ sexual identity was known, on average, by 75% of the listed figures. While this represents a high percentage–especially compared to the percentage demonstrated in other life stages–it nonetheless indicates that Italian LGBQ+ people struggle to be completely disclosed in all relationships and contexts.

To obtain more detailed information and to gain greater comprehension of the CO process, we investigated whether–and at what age–participants had CO to a series of significant figures (D’Augelli and Grossman, 2001; Jenkins Morales et al., 2014; Pistella et al., 2020a). To extend prior research on this topic, we included in our study a set of figures who had not been considered previously, including nephews, grandparents, and neighbors. In line with the results of previous studies, the present study found that most participants first CO to their best friend (Baiocco et al., 2012; Costa et al., 2013; Salvati et al., 2018; Pistella et al., 2020a). Within the family, participants were more likely to CO first to siblings than to parents, confirming the important role played by siblings in the CO process within the family context (Pistella et al., 2020a). Grandparents were less commonly disclosed to, perhaps as a result of participants’ desire to protect their grandparents from any negative consequences of disclosure. Indeed, previous studies have found that LGBQ+ grandchildren perceive their grandparents as fragile, and that the experience of CO to grandparents is often mediated by other family members (Scherrer, 2016).

Among the figures to whom LGBQ+ people CO to later are children and nephews, followed by neighbors, employers, family doctor, and coworkers (D’Augelli and Grossman, 2001). However, the present findings concerning the average age of CO to important figures must be interpreted with caution. For instance, older adults had CO to their grandparents at a younger age than the age at which they had CO to their best friend, father, and mother. But if we look at the percentage of disclosure, only four older adults (12%) had CO to their grandparents and only eight older adults (18%) had CO to their father, whereas almost all (92%) had CO to their best friend and almost half (47%) had CO to their mother. Consequently, it is not possible to rigorously compare the average age of first CO to significant figures between the participants and groups.

Older adults emerged as the least disclosed group to almost all relevant figures, with the exception of the family doctor (Jenkins Morales et al., 2014). The greater tendency of the older generation to conceal their sexual identity can be interpreted as a consequence of the stigmatized historical period in which they grew up, when non-heterosexual behavior was considered immoral and/or condemned by all institutions (e.g., religious, legal, and medical institutions) (Shankle et al., 2003; Rosati et al., 2018). Moreover, although they CO at a later average age than both other groups, their decision to conceal their sexual identity from their parents might be due to the fact that their parents passed away before they became comfortable disclosing. The exception of the family doctor, to whom older adults presented the highest rate of disclosure, can be explained by the greater need of LGBQ+ older adults to access healthcare services; this might entail greater confidence in the family doctor, including confidence in disclosing one’s sexual identity (Gardner et al., 2014; Rosati et al., 2020).

No differences were found between generations in levels of minority stressors. This result contrasts with the findings of previous studies, which have assumed aging LGBQ+ people to be more stigmatized than emerging adults (Rosenfeld, 1999; Shankle et al., 2003; Dentato et al., 2014). However, such studies have generally been based on a single age group (e.g., older adults), and have thus inferred differences without the supporting evidence of empirical comparisons of cohorts. It is likely that shifts in the historical environment impact experiences of sexual minority identity, and that other important factors may contribute to determining LGBQ+ peoples’ experiences of minority stress (Frost et al., 2019). For instance, although older adults grew up in a more cis-heterosexist and cis-heteronormative social context than young adults, they had also had a greater opportunity to process life experiences and elaborate on aspects of their sexual identity, to gain resiliency (Kimmel, 2015). Moreover, the greater tendency of LGBQ+ older adults to conceal their sexual identity probably protected them from experiences of discrimination and harassment (D’Augelli et al., 1998; D’Augelli and Grossman, 2001; Jenkins Morales et al., 2014).

Although Italy is a very religious country, to our knowledge, no previous studies have investigated the effect of CO in Italy within a religious context. Moreover, there is a lack of information in the psychological literature on the relationship between CO in religious contexts and levels of minority stress. Given the significant relationship between minority stress and the quality of one’s social context, and considering that cis-heterosexist assumptions generally characterize Catholic environments, we expected to find higher levels of minority stress among participants who were both Catholic and openly LGBQ+ within their religious community. Among the Catholic participants in our sample, approximately 67% had CO to their religious community; this figure is higher than the percentages reported in previous research (Legate et al., 2012). Furthermore, older adults (84%) were significantly more disclosed to this community than were young adults (48%). We can infer that the need to integrate religious aspects with one’s LGBQ+ identity may be more relevant for older adults, who in fact have been found to be significantly more religious than young adults. As a result, older adults in our study may have CO to their religious community in an attempt to live more authentically in the context of the Catholic Church.

More than half of the participants who had CO to their religious community were not accepted by that community. The fact that older adults were the most rejected group may suggest a decrease in cis-heterosexist attitudes and sexual prejudice within Catholic institutions in Italy. However, given that we did not evaluate the cis-heterosexist attitudes and sexual prejudice and the information about the age of CO with the Catholic community was missing, this explanation is only speculative. Our thematic analysis (Braun and Clarke, 2006) uncovered that non-acceptance from the Catholic community could manifest in subtle or open forms of rejection. Subtle rejection was expressed as an *invitation to change* participants’ sexual orientation, whereby participants’ behavior was condemned, but not their personhood, as transmitted by the message “hating the sin but not the sinner” (Valera and Taylor, 2011). In such situations, LGBQ+ people experienced the paradox of being accepted, on the condition that they recognized that their non-heterosexual behavior was wrong (Severson et al., 2014). *Open rejection* was characterized by a condemnation of LGBQ+ identities and behaviors, often accompanied by the administration of coercive techniques aimed at “correcting” participants’ sexual identity. Among the psychological consequences reported by participants who received such open rejection were guilt, inadequacy, mistrust, humiliation, and depression, thus confirming the highly detrimental effects of such reactions on mental health (American Psychological Association, 2009).

As hypothesized, CO to the Catholic community was associated with a higher level of minority stress. Specifically, participants who had CO to their religious community experienced more discrimination, vigilance, and ISS than those who remained concealed or who did not belong to the Catholic Church. As shown by Legate et al. (2012), disclosure can have a negative impact on mental health if it occurs within an unsupportive context; therefore, concealment could mitigate the impact of stress under certain circumstances (Cole, 2006). Similar to what found by previous authors (D’Augelli et al., 1998; D’Augelli and Grossman, 2001) in family and unspecified contexts, the present study found that, the more LGBQ+ people had disclosed their sexual identity to their religious community, the more at risk they were of experiencing discrimination and harassment. Participants likely considered their experiences of the Catholic community’s negative reactions to their CO as discriminatory.

The present study also found that participants who were more disclosed to their religious community experienced greater vigilance—a state of constant alert related to the fear of being a target of prejudice (Crocker et al., 1998). In line with Meyer’s (2003) theorization, minority stressors are overlapping and interdependent, so that “the greater one’s perceived stigma, the greater the need for vigilance in interactions with dominant group members” (p. 680). Therefore, we suggest that LGBQ+ participants who had CO to their religious community experienced greater vigilance as a consequence of the greater discrimination they experienced from the Catholic Church.

Finally, LGBQ+ people who had not CO to their religious community presented higher levels of ISS, confirming the potential harmful impact of religiosity in LGBQ+ self-perception (Barnes and Meyer, 2012; Lingiardi et al., 2012; Severson et al., 2014; Sowe et al., 2014; Nardelli et al., 2020). ISS entails an insidious process of self-stigmatization that leads to self-devaluation and internal conflict. It has been shown to have several negative consequences on mental health, including depression, anxiety, substance abuse, self-harm, and suicidality (Williamson, 2000; Meyer, 2003; Kuyper and Fokkema, 2011; Lehavot and Simoni, 2011). The present study also found that, the older the participant, the more likely they were to have experienced discrimination and vigilance in the context of CO to the Catholic community. No significant age effect was found for ISS.

### Limitations and Directions for Future Research

The present study is not exempt from limitations, which should be considered when designing future research. First, we only focused on generational differences in CO experiences, and did not consider variation in participants’ gender and/or sexual orientation. We also acknowledge that relevant differences exist among subgroups of the LGBQ+ population, which should be investigated in future studies through larger samples. Additionally, future research should also consider the specific CO experiences of transgender and non-binary people. Second, not all participants had been able to CO to all of the significant figures examined because, for instance, they were lacking one or both parents, siblings, and grandparents when they became aware of their sexual orientation. Therefore, although we differentiated participants who had not CO by choice from those who had not CO for lack of opportunity, this limitation may still impact the comparison between groups on the rate of CO to specific figures.

Finally, given the qualitative nature of the open-ended question concerning the quality of the reactions of the religious community to participants’ CO, as well as the small number of participants (*n* = 50) who experienced such reactions, we could not verify whether minority stressor experiences related to the quality of the community reactions, rather than to CO, *per se*. More generally, although data from the present study may be valid for numerous reasons, they are not generalizable to all Western countries, nor can they fully explain the complex relationship between religiosity, sexual minority identity, and the CO process. This study should therefore be contextualized in the Italian context, where the Catholic religion plays a significant role in both historical and contemporary cultural traditions, and is embedded in the society’s shared belief system.

## Conclusion

The CO process is fundamental for identity integration among sexual minorities. However, the effects of CO can vary greatly depending on contextual variables, such as the historical period in which people grow up, as well as the social contexts they frequent. By considering three generations of Italian LGBQ+ people, this study analyzed meaningful features of CO in family, social, and religious contexts. This study contributed to expand knowledge about stress processes related to sexual minority identity and CO. We found that different generations of sexual minorities differ in the CO process, even if they are similar in the level of minority stressors. Our findings confirmed that CO is a non-linear process, rather than an event, which can increase or protect from stress, based on contextual factors.

## Implications

The empirical findings on the differences and similarities between diverse generations of LGBQ+ people can help mental health professionals implement targeted interventions on the basis of priority needs for each age cohort. Moreover, our results suggest that mental health professionals should pay particular attention to clients from cultural or religious traditions that are less accepting of sexual minorities, as such clients may experience intense struggles when attempting to integrate their sexual and religious identities (Baiocco et al., 2018). Although religious belief is generally associated with positive psychosocial outcomes, CO to one’s religious community may increase minority stress, thus confirming that the impact of CO on wellbeing is strongly connected to the quality of the environment in which it occurs (Legate et al., 2012). It is important that psychotherapists and psychologists reflect on the potentially detrimental effect of CO within an unsupportive context (together with their LGBTQ+ clients), rather than encourage CO *tout court*. Further, clinicians should embrace a complex understanding of the multiple individual and social factors involved in sexual identity development and affirmation (e.g., self-awareness, authenticity, social support, and community connectedness) (Riggle et al., 2014), rather than focus on the CO process as the only viable means for LGBQ+ people to achieve self-acceptance (Rosenberg, 2018).

As per the social implications of this research, our findings contribute to give visibility to LGBQ+ Catholic people, highlighting their personal experiences through qualitative analysis and the challenges they face in crossing Catholic contexts. LGBQ+ Catholic people risk being isolated both in the LGBTQ+ community and within their religious community, or to conceal some crucial aspects of their identity (e.g., sexual or religious identity) in order to feel accepted and integrated in such communities (Beagan and Hattie, 2015). Efforts would be needed from both LGBQ+ organizations and grassroots Catholic groups to foster an inclusive environment and to counter discrimination and stigma. Other Christian religions in Italy have already started working in this direction, through the creation of support groups, training sessions, and experiences-exchange meetings, which can be used as a reference or good practice.

## Data Availability Statement

The raw data supporting the conclusions of this article will be made available by the authors, without undue reservation.

## Ethics Statement

The studies involving human participants were reviewed and approved by Ethics Commission of the Department of Developmental and Social Psychology of Sapienza University of Rome. The patients/participants provided their written informed consent to participate in this study.

## Author Contributions

FR conducted the data collection for the study and wrote the manuscript. JP and RB collaborated with designing and writing the study, and together with FR conducted data analysis. MN collaborated in writing and editing the final manuscript. All authors read and approved the final manuscript.

## Conflict of Interest

The authors declare that the research was conducted in the absence of any commercial or financial relationships that could be construed as a potential conflict of interest.
